# Successful treatment of mucoepidermoid carcinoma in the left main bronchus

**DOI:** 10.1186/s40792-015-0087-4

**Published:** 2015-09-22

**Authors:** Osamu Kawano, Daisuke Yuki, Ichiro Fukai, Noriaki Tsubota

**Affiliations:** Department of General Thoracic Surgery, Suzuka General Hospital, 1275-53 Yamanohana, Yasuzukatyou, Suzuka, Mie 513-0818 Japan; Department of Thoracic Oncology, Hyogo College of Medicine, 1-1 Mukogawatyou, Nishinomiya, Japan

**Keywords:** Tracheobronchial disease, Thoracotomy, Bronchoplasty, Mucoepidermoid carcinoma, Bronchial reconstruction

## Abstract

Here, we report the successful treatment of a 40-year-old man with mucoepidermoid carcinoma that originated in the proximal end of the left main bronchus close to the carina. He underwent wide and deep airway wedge resection, including the distal trachea and part of the carina via left postero-lateral thoracotomy. He has demonstrated neither anatomic complications nor disease recurrence 2 years after the operation.

## Background

A low-grade malignant tumor in the large airway is a good indication for one-stoma-type bronchoplasty, which does not necessitate sacrificing of the parenchyma [1]. However, the ideal surgical approach for a tumor involving the carina is controversial. Here, we report a case of a mucoepidermoid carcinoma that originated in the proximal end of the left main bronchus close to the carina that was managed via left postero-lateral thoracotomy.

## Case presentation

**Fig. 1:**
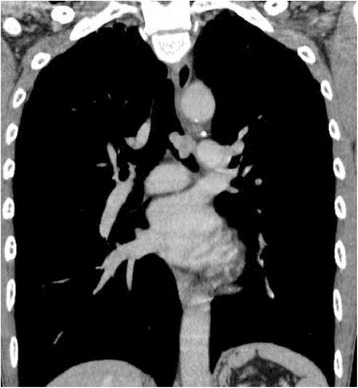
CT with multiplanar reconstruction revealed a polypoid tumor originating at the lateral and distal part of the trachea and protruding into the airway

**Fig. 2:**
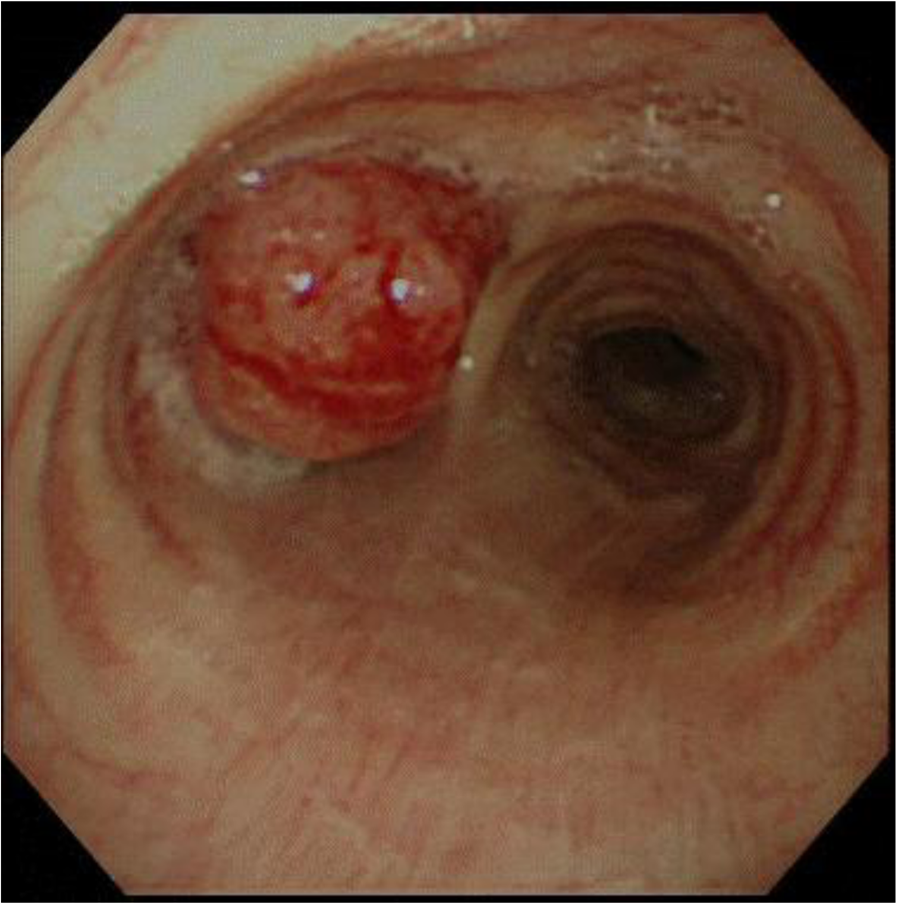
The bronchoscopy revealed the polypoid tumor protruding from the left main bronchus into the carina

A 40-year-old man, complaining bloody sputum and a non-productive cough, was referred to our hospital. Computed tomography (CT) with multiplanar reconstruction revealed a 25-mm polypoid tumor that had originated in the lateral and distal portion of the trachea and protruded into the airway (Fig. 1). Under general anesthesia, bronchoscopy showed a polypoid tumor, obstructing the orifice of the most proximal portion of the main bronchus, and the tumor was temporarily resected via snaring to relieve dyspnea, prior to radical surgery (Fig. 2). The tumor was diagnosed as a mucoepidermoid carcinoma.

**Fig. 3:**
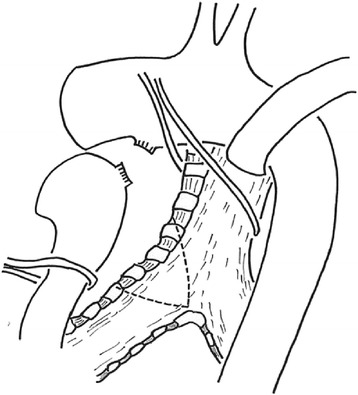
*Dotted line* indicates a resection line including lateral side of proximal end of the main bronchus and lower end of the trachea

To expose the left main bronchus and the carina, left postero-lateral thoracotomy through the fourth intercostal space was selected. The intraoperative ventilation was performed with the double-lumen endobronchial tube for the right bronchus. Accessing the carina was possible by bringing it down toward the operator below the aortic arch by encircling the trachea with traction tape. While encircling the trachea, the part of azygos vein were exposed, but SVC were not exposed. #10R and #4R lymph nodes were not dissected. After Botallo’s ligament was divided, the aortic arch was retracted upward and the pulmonary artery was retracted downward to create better exposure. The tumor growing outside of the bronchial cartilage was resected via deep wedge and wide resection, including the trachea and part of the carina (Fig. 3). Trachea incision was on more peripheral side than the tracheal cuff of the endobronchial tube. An anastomosis was performed by tying three stitches inside the airway at the deepest part, which allowed for easy handling of the sutures. Frozen section analysis showed that the resection margins were free of residual tumor cells. Hilar lymph node and mediastinal lymph node (#10L and #4L) dissection was then performed. Pathological examination revealed that the tumor was completely resected, and no lymph node metastasis was detected. The patient is alive and healthy with no recurrence 2 years after the operation.

### Discussion

Low-grade malignant tumors of the bronchial airway are often managed via circumferential or wedge bronchial resection, because they require minimal surgical margins [2]. Such neoplasms that originated in the proximal left bronchus close to the carina and trachea have been the subject of various discussions on surgical approaches. Right thoracotomy and median sternotomy are common approaches for carina resection and reconstruction [3]. Through these approaches, proximal left main bronchial resection extending beyond the carina and trachea would be possible. However, when the lesion exists at the distal end of the left main bronchus, these approaches would cause inadequate margin along the distal resection line and inadequate left hilar lymph node sampling. Left thoracotomy accompanied by encircling of the trachea with traction tape, with or without resection of Botallo’s ligament, would provide better access to the carina and the distal end of the left main bronchus, whereby an excellent operative field for anastomosis and lymph node dissection could be obtained [4]. If needed, left thoracotomy can provide additional distal airway mobility by using the pericardial hilar release technique. Our procedure will be suitable for mucoepidermoid carcinoma but not for adenoid cystic carcinoma, because it extends longitudinally, which often results in additional wide resection.

Knotting inside the airway makes anastomosis easier, especially in cases of a deep operative field. Here, the three knots placed inside of the airway at the anastomotic site did not cause any airway trouble during the postoperative course. This technique would be more reliable in cases of a deep operative field in the mediastinum and in cases of a more complicated anastomosis.

## Conclusions

Bronchoplasty without sacrificing of the parenchyma for a low-grade malignant tumor that originated in the main bronchus is an effective operation. However, it is necessary to consider before surgery, such as approaches and surgical procedures.

## Consent

Written informed consent was obtained from the patient for publication of this case report and any accompanying images. A copy of the written consent is available for review by the Editor-in-Chief of this journal.
